# Consent and its discontents: the case of UK Biobank

**DOI:** 10.1007/s11019-025-10276-5

**Published:** 2025-07-17

**Authors:** Gulzaar Barn

**Affiliations:** Department of Philosophy, University of Amsterdam, PO box 94201, 1090 GE Amsterdam, The Netherlands

**Keywords:** Biobank, Consent, Dual use, Genetic enhancement

## Abstract

UK Biobank is a major biomedical database and research resource, holding the genetic, health, and lifestyle information of half a million adult volunteers. Its datasets are accessible to approved researchers from academic, charity, government, and commercial organisations for health-related research in the public interest. Drawing upon a range of approved projects and the downstream applications of this research, I suggest that UK Biobank datasets have been processed towards ends that are inimical to its stated aims, breaking the terms of consent under which its participants entered the study. First, I provide an overview of the broad consent model employed by UK Biobank in recruiting participants and using their data. The consent documents and participant information leaflets used exhibit information failures in their framing of health-research in terms of disease and treatment, obscuring the full range of lawful uses of participants’ data. Beyond this, certain approved uses of UK Biobank data, including studies by insurance companies and direct-to-consumer genetic testing companies, arguably fall outside UK Biobank’s stated aims altogether. Moreover, UK Biobank has not adequately safeguarded against “dual use” issues. Tracking the trajectory of research outputs that used biobank data, I suggest that approved uses of biobank datasets have gone on to have objectionable further applications that are not in the public interest. Such applications include the development of polygenic scores that seek to predict “intelligence” for use in commercial embryo screening services. Such tools are rife with risk of harm and are being deployed without sufficient public deliberation or oversight.

## Introduction

UK Biobank is a biomedical database and research resource, holding the genetic, health, and lifestyle information of half a million UK adult volunteers. It was established by funding from the Wellcome Trust, Medical Research Council, Department of Health, Scottish Government and the Northwest Regional Development Agency (UK Biobank 2024). Its funding sources have since expanded to include former CEO and Chairman of Google, Eric Schmidt, and Ken Griffin, founder and CEO of the hedge fund, Citadel (Great Britain. Department for Science, Innovation & Technology 2023).^1^ The large datasets held by UK Biobank happen to be highly commercially valuable sources for training machine learning algorithms.^2^ Set up as a resource to facilitate discoveries that aim at improving public health, UK Biobank aims to enable research into the genetic component of disease. The project generated scientific criticism from the outset. Helen Wallace, of the organisation, GeneWatch UK warned that UK Biobank’s aims were controversial and that the prediction of future common illnesses by testing people’s genetic make-up is unlikely to be a successful or cost-effective means of disease prevention (Wallace 2005). Wallace also argued that this focus on individual genetic susceptibility to disease was continuous with a “scientific bandwagon” generated by the tobacco industry, which first began to use scientific research to promote the idea that a minority of smokers are ‘genetically predisposed’ to lung cancer in the 1950s (Wallace 2009). By focusing on internal, biological risk factors for diseases such as cancer and heart disease, the tobacco industry aimed to detract attention from its products. As a result of its lobbying and funding of university research institutes, genetics came to dominate the cancer research agenda.

The wider scientific community also raised concerns with UK Biobank prior to its establishment (House of Commons - Science and Technology - Appendices to the Minutes of Evidence 2003; Barbour 2003). Some scientists with intimate knowledge of the project and its aims had concerns with the design, preferring standard case-control studies (Clayton and McKeigue 2001). In a 2009 review of UK Biobank, GeneWatch UK highlighted the scientific consensus that genes are thought to be poor predictors of common diseases like cancer in most people (Wallace 2009; Baker and Kaprio 2006; Buchanan et al. 2006a, b; Terwilliger and Weiss 2003). Indeed, some scientists warned that undertaking ever larger studies is a waste of money and that misleading promises about the predictive power of genes are being made in order to secure research funding (Buchanan et al. 2006a, b). An initial report from the Medical Research Council suggested that UK Biobank was politically driven and that it had been funded before the scientific case for it had been properly established (UK Parliament 2003).

Some of these critics rescinded their disapproval, suggesting that the biobank could indeed be worthwhile if it were conceived as an epidemiological project about how diseases develop, without a focus on genetics (Jha 2006). UK Biobank has maintained a focus on researching the “genetic determinants of a wide range of health outcomes”, however, and its whole genome sequencing data on half a million participants is the biggest whole genome dataset in the world (UK Biobank 2018). In addition to cryogenically stored biological samples, UK Biobank holds lifestyle information such as participants’ educational attainment, marital status, ethnic background, and the results of cognitive tests it has conducted. The database is regularly expanded with additional information, but not all participants are equally as responsive to the supplementary surveys, which may lead to sampling issues. Indeed, UK Biobank has been criticised for its selection bias in general (van Alten et al. 2024; Schoeler et al. 2023). It relies on volunteers, but those who choose to participate report being in better health in relation to national averages, which complicates the ability to derive scientifically valid estimations from their data. Information from participant surveys is linked with participants’ “health-related records to provide a deeper understanding of how individuals experience diseases” (UK Biobank 2015). These health records have recently come to include GP primary care data, which UK Biobank can now access directly, bypassing GP and participant approval (Flynn 2024). The idea is that these de-identifed datasets are accessable to approved researchers from academic, charity, government and commercial organisations in order to conduct health-research in the public interest.

I introduce these concerns, regarding possible commercial interests and validity of the study design, in order to set them aside. Granting the terms under which UK Biobank operates, I would like to suggest that by its own lights, it has not met certain conditions of operation centred on informed consent and conducting research in the public interest. That is, UK Biobank data has been processed towards ends that may be inimical to the “health and wellbeing of society” (UK Biobank 2024). I draw attention to various controversial uses of UK Biobank data and highlight the systematic potential for such continued misuse. Even if UK Biobank were to make the case that these controversial uses do indeed fall within the public interest, the ethical concern regarding the validity of participant consent remains. My analysis suggests that participants are likely to understand their participation in the project primarily in terms of its role in researching disease and treatment, rather than in such controversial uses. In not understanding the fall range of lawful uses of their data, including these unexpected and controversial uses, participants’ consent may not have been meaningful. Approved uses of UK Biobank datasets include (1) research into the genetic basis for non-disease traits such as height and cognitive ability which has since been commercially applied in the screening of embryos during IVF procedures by reproductive clinics (UK Biobank 2015; UK Biobank 2023), (2) direct-to-consumer genetic ancestry companies such as 23andMe (UK Biobank 2022; UK Biobank 2021; UK Biobank 2017; UK Biobank 2022; UK Biobank Research 2024), and (3) insurance firms training their risk prediction algorithms (UK Biobank 2022; UK Biobank 2023; UK Biobank 2020).

I begin by providing an overview of the broad consent and “trust” model employed by UK Biobank in recruiting participants and using their data. Participants give their broad consent to participate in an *area* of research, and UK Biobank relies upon ‘legitimate interests’ and ‘reasons of public interest’ as its lawful bases under GDPR for *processing* the data obtained from them. While participants could not have provided “informed consent” to future, unknown uses of their data, the language of consent is employed by UK Biobank in the initial grounds for participation in the project. I query the extent to which this preliminary consent condition has indeed been met. In various participant information documents, UK Biobank frames third-party access to data primarily in terms of “health-research”, which is expanded and clarified by examples that centre on disease and treatment. This focus obscures other seemingly lawful but morally objectionable uses of participants’ data and thus, to some extent, disturbs meaningful consent. Next, I suggest that certain uses of the data – such as by insurance and direct-to-consumer genetic testing companies – arguably fall outside UK Biobank’s stated aims and therefore break the terms of consent, even if it were valid. Next, I outline another safeguarding problem: the issue of dual use. I claim that certain uses of data may initially present as satisfying UK Biobank’s data use-legitimating grounds centred on health research in the public interest but go on to have an objectionable further use. This is the case with research into genes and cognitive ability that has contributed to the subsequent creation of polygenic scores for “intelligence” in the context of embryo screening services. Such tools are scientifically contested (Karavani et al. 2019), display links with eugenics and race science (Kevles 1995), and are being deployed without sufficient public oversight. Such considerations place this research outside of the public interest, calling into question the moral legitimacy of relying on broad consent: many participants are unlikely to consent this specific use of their data. Indeed, such unforeseen but harmful uses arguably break the terms of consent altogether, which, I have suggested, are already predicated upon a misleading understanding of “health-related research”. Dual use issues such as these trouble the permissive data access policy under GDPR and point to concerns with the efficacy of UK Biobank safeguards. The fast-paced nature of genetic research and the commercial interests that accompany it may facilitate and proliferate such dual use issues, suggesting that more action is required by UK Biobank, such as the greater restriction of research.

## Consent, GDPR, and legitimate interests

### Informed consent and broad consent

Informed consent is a key pillar in biomedical and research ethics (World Medical Association 2024), often viewed as a legitimacy requirement (Beauchamp and Childress 2001; Miller and Wertheimer 2009). That is, consent makes certain actions permissible, and its absence may reconstitute those same actions as harmful violations, transforming “a trespass into a dinner party; a battery into a handshake; a theft into a gift; an invasion of privacy into an intimate moment” (Hurd 1996). Consent is the basis of *participation* in UK Biobank (i.e. having your materials and data stored in the database), but it is not the legal basis on which UK Biobank *processes* the data collected. Instead, UK Biobank relies upon “legitimate interests” and “public interest in the area of public health and for scientific research purposes” under UK GDPR as the primary lawful bases for data processing (“Basis of your participation - Privacy Notice for UK Biobank Participants,” 2025). Further, the consent sought by UK Biobank is “broad” and given to an *area* of research. It is not possible for such consent to be “informed” in the standard sense due to the evolving nature of the resource and the fact that uses of the data are unknown at the time of consent. Even so, I suggest that this preliminary consent condition grounding participation in UK Biobank is not met due to the incomplete way in which UK Biobank’s stated purpose is defined and explained to participants. Further, I make the case that certain uses of biobank data, such as by insurance companies, may fall outside the stated aims of UK Biobank altogether, and therefore break the terms of consent. This may serve to ground a formal objection by the participants (“data subjects” under GDPR), accompanying withdrawal from the project.

Biobanks differ from traditional human research projects where one-off consent is given to participate in a specific study with a discretely defined aim. By contrast, UK Biobank holds participants’ data in a database and permits access to researchers for projects that are evolving and unforeseen at the time of consent. Ethicists such as Heather Widdows, Emma Bullock, and Sean Cordell were early critics of the language of consent in the context of biobanks. They highlighted the epistemological concern that the nature of the future research projects was opaque to both UK Biobank and participants, which necessarily precludes the possibility of “informed” consent (Widdows and Cordell 2011; Bullock and Widdows 2011). Further, the informed consent model is individualistic and cannot respect the interests of third parties that are implicated in the donation of genetic materials and biobank research. Genetic information necessarily involves data about non-consenting genetic relations, such as family and wider population groups, for example (Bullock and Widdows 2011; Widdows 2013).

Broad consent was plausibly favoured for pragmatic reasons centred on the prohibitive costs of re-contacting 500,000 participants for their consent to specific research projects that were to draw upon their data. Participants have a right to ‘withdraw from UK Biobank for any reason at any time’ (“Basis of your participation - Privacy Notice for UK Biobank Participants,” 2025), which may serve to mitigate some concerns with the broad consent model. However, such a right cannot retroactively offset the harms associated with any wrongful processing of data that has already occurred. Participant data cannot be removed *post hoc* from studies that have already drawn upon such data, or from datasets that have already been accessed and continue to be held by researchers. It should also be noted that the right to withdraw is only available to competent informed adults: UK Biobank retains the right to store and control the data of those who may have lost capacity since its inception. As UK Biobank participants were recruited in mid to late life, aging, death, and cognitive decline are inevitable, thus vitiating their ability to withdraw consent. This in itself is an ethical quandary that cannot be explored here.

Most fundamental to the questions surrounding participant understanding addressed in this paper, a meaningful right to withdraw depends upon participants being continuously alert to the research projects that draw upon UK Biobank data and appraising their value. Approved research projects are made publicly available *ex post* on a database and “potentially contentious” (UK Biobank 2024) cases go through a form of ethics approval prior to this.^3^ These safeguards supplement the broad consent model, whereby subsequent uses of participant data are seen as justifiable without the need to return to the participant each time for their approval (McHale 2022). This contrasts with specific or continuing consent, where individual consent would be obtained for each research project, reflective of the idea that a person’s views as to what forms of scientific research are legitimate may change over time (McHale 2022). Such ongoing consent was apparently favoured in the original consultation exercises for UK Biobank, where some members of the public and other stakeholders believed that individuals should be given the opportunity to opt out of individual studies that didn’t align with their values (McHale 2022; UK Biobank 2004).

UK Biobank employs the language of consent in its Privacy Notice for UK Biobank participants, Ethics and Governance Framework (EGF), consent forms, and participation information leaflets. The EGF states that “consent will be sought to participate in UK Biobank.” It further stipulates that as “it will be impossible to anticipate all future research uses, consent will be sought for research in general that is consistent with UK Biobank’s stated purpose (rather than for specific research).” The UK Biobank’s stated purpose is “to set up a resource that can support a diverse range of research intended to improve the prevention, diagnosis and treatment of illness, and the promotion of health throughout society” (UK Biobank 2006). The EGF assures that “further consent will be sought for any proposed activities that do not fall within the existing consent” (Laurie 2009). After UK Biobank adopted this broad consent model, Heather Widdows highlighted that such an approach would only work if the proposed safeguards to supplement the consent were effective. Participants were trusting UK Biobank to “not misuse their samples and data” (Widdows 2013). Widdows cautioned that the strength of the trust model therefore lay in the robustness of the additional ethics and governance mechanisms (Widdows 2013). These mechanisms are primarily the EGF and the Ethics and Governance Council (EGC). The EGC was once an independent guardian of the EGF, but it has since been replaced by an in-house Ethics Advisory Committee (EAC) of the UK Biobank Board (UK Biobank 2024) It had been the role of the EGC to ensure that research undertaken using the biobank resource was consistent with its aims and purposes, and in the public interest. Now, an in-house Access Committee is responsible for making the key decisions about scientific access to the resource, “notably those regarding the use of depletable samples or potentially contentious research” (UK Biobank, 2024). Later in this section, I will suggest that these safeguards seem to exhibit flaw in that some approved access may fall outside UK Biobank’s stated purpose. First, however, I consider that the initial consent to participate may not have been meaningful.

It is important, at this point, to anticipate the following rejoinder from UK Biobank. Legally, participants provide consent for the use of their biological material for research purposes. However, as aforementioned, the processing of their data is not carried out on the basis of consent. Data is processed according to ‘legitimate interests’ and ‘reasons of public interest’, including public health and scientific research. *Legally*, it may not matter, therefore, if participants fail to understand the full range of lawful uses, as their consent is not necessary for such processing. The consent obtained for participation is not intended as legal consent under data protection law, but rather as ethical consent in accordance with research norms. Despite this legal distinction, however, UK Biobank consent documents use language that may lead participants to believe they are giving meaningful consent to the use of their personal data in research.^4^ The privacy notice on the website clears this up, but the consent documents remain somewhat ambiguous having been written prior to GDPR and the grounds for processing that it introduced. The above rejoinder fails to extinguish the concerns I outline, therefore. Firstly, it fails to acknowledge that legal and ethical concepts of consent remain blurred the consent stage. Participants may be unclear on the legal authority they provide – they do not in fact provide legal consent for the research use of their personal data but may believe that they do. Secondly, the ethical consent to participate may not have been valid at all, which may or may not have further legal implications but certainly raises moral concerns. Below, I explain why this ethical consent fails to be valid, centred on the information failures surrounding the full lawful use of participants’ data.

To attend to this latter question, is instructive to engage in some conceptual analysis of consent. Consent only exhibits its morally transformative force when it meets certain conditions. Alan Wertheimer’ influential account holds that certain background defects can invalidate consent, such as coercion, lack of competence, and deception. Coercion is often understood as involving a threat to make someone worse off than their existing baseline, or worse than they have some right to be. A lack of competence typically refers to being below a certain age or lacking appropriate mental capacities (Wertheimer 2003). There is no reason to think that either of these defects are present in the case of participants’ initial consent to UK Biobank. Wertheimer’s third background defect, deception, however, can involve failures to disclose important information. Due to the presentation of the biobank as a resource primarily associated with ‘health research’, where health is explained to participants in terms of *treatment for disease*, commercial and non-disease uses of participants’ data may not have been appropriately outlined by UK Biobank or sufficiently understood by participants. Through an analysis of the consent documents, information leaflet for participants, and general website information displayed by UK Biobank, I suggest that a sufficiently representative outline of viable uses is lacking. As a result, participants are unlikely to fully comprehend the breadth of possible data applications and may not foresee various uses that may be lawful but do not constitute treatment for disease. The validity of their consent is therefore called into question.

Wertheimer offers his account in the context of sexual relations, considering whether fraud or misrepresentation between lovers invalidates consent. Deception is said to undermine the moral and legal transformative power of consent as “it precludes B from being able to decide whether engaging in sex with A is in her interests or compatible with her values” (Wertheimer 2003). The questions as to what precisely constitutes deception, concealment, and sufficient information disclosure remain open, however, and cannot be settled here. Onora O’Neill supplies a critical intervention in suggesting that while the importance of consent lies in the reasonable assurance it provides that a patient has not been deceived or coerced, “genuine consent is not a matter of overwhelming patients with information, arrays of boxes to tick or propositions for signature” (O’Neill 2003). Rather, it is exhibited where “patients can control the amount of information they receive, and what they allow to be done” (O’Neill 2003). While UK Biobank participants may not have been presented with exhaustive information about all possible uses of their data, this need not result in informational failures that invalidate consent. If participants were in some sense able to control the amount of information they received, however, this might be seen as sufficient information disclosure. Whether such control has been exhibited will be adjudicated upon later in this section.

The broad consent given by participants was to an *area* of research, specifically, health research. This is indicated in the following manner in the consent form:

The purpose of UK Biobank is to set up a resource that can support a diverse range of research intended to improve the prevention, *diagnosis and treatment of illness*, and *the promotion of health throughout society*.^5^

I give permission for access to my medical and other health-related records, and for long-term storage and use of this and other information about me, for *health-related research purposes* (even after my incapacity or death).^6^

I give permission for long-term storage and use of my blood and urine samples for *health-related research purposes* (even after my incapacity or death), and relinquish all rights to these samples which I am donating to UK Biobank.^7^

These three quotes are the most pertinent to the disclosure of information surrounding UK Biobank’s research aims, and therefore to the informed consent to participate in the project. Notably, each statement places focus on *health* as a guiding objective. While the first quote includes “the promotion of health throughout society” as an aim separable from the “diagnosis and treatment of illness”, it is plausible to opine that participants may be disposed to view these aims as interchangeable and assume that all research ‘promoting health’ is to do with disease, given there is no separate definition provided of the former conception. The aim of ‘promoting health’ is broad, and the lack of specificity may lead to its conflation with disease-associated understandings of health.

The participant information leaflet, stipulates the following:

Information and samples from UK Biobank participants will be available only to researchers who have relevant scientific and ethics approval for their planned research. This could include researchers who are working in other countries and in commercial companies *looking for new treatments* (UK Biobank 2010).

This sentiment is echoed in the “FURTHER INFORMATION LEAFLET (supplementing the main Information Leaflet)”:

Pharmaceutical and other health-based companies will be able to access the study data for approved research, as this may help in the *identification of new treatments* (UK Biobank 2009).

These quotes demonstrate that the commercial use of the data is communicated from the outset. However, both documents outline this commercial use in terms of *treatment for disease*, which does not cover the full range of permitted research access, and may reinforce a misleading, exclusionary focus on health-research as understood in this restricted way. These statements, individually and jointly, imply that companies accessing the data are doing so to create new treatments. While this inference may characterise the *primary* use of UK Biobank data by various *pharmaceutical* companies, it certainly does characterise *all* lawful uses of data. The examples included – namely, the discovery of new treatments – are plausibly foreseeable and intentional. Indeed, further such commercial uses of the data could have been included from the outset and perhaps should now be provided to update the consent documents, enabling participants to meaningfully re-evaluate their involvement.

This is because pharmaceutical companies are not the only companies accessing the data. Various DNA ancestry companies, including the now bankrupt 23andMe, which offer direct-to-consumer genetic testing have accessed UK Biobank data in order to refine their genomic prediction services. In general, direct-to-consumer genetic testing companies have been criticised for their inaccurate and unreliable tests (Tandy-Connor et al. 2018), failing to safeguard against psychological effects of testing (Broady et al. 2018), and their data-sharing practices (Hendricks-Sturrup and Lu 2019). Having recently filed for bankruptcy, 23andMe stated it would pursue a sale of the company, which would including selling its consumers’ data (Duffy 2025). New ownership could generate changes in how the data is stored and the purposes it is used for. UK Biobank datasets are also implicated in this transaction: the sale of assets plausibly includes the tools 23andMe developed that drew upon UK Biobank participant data, which the company accessed twice in 2022 (UK Biobank, 2022; UK Biobank, 2022). In light of such long-standing and predictable profit-sharing and data privacy concerns, participants to UK Biobank might question the extent to which such companies’ activities are in the public interest in the first instance, an issue to be explored later in this section.

Returning to Wertheimer’s analysis of deception as invalidating consent, there is a sense in which the treatment-based framing in the leaflet and consent forms, involves failures to disclose important information surrounding other commercial use. As a result, participants may believe that commercial use outside of treatment development is not permitted. On O’Neill’s analysis of consent and informational control, it seems that the tiered control of information has not evaded this issue. The kind of control of information that is pertinent here would have been unattainable due to the specific nature of UK Biobank as a resource for research that is unknown at the time of consent. O’Neill states that patients could be given control over the amount of information they choose to receive, by offering easy access to more specific information that lies behind an initial, or second, or third layer of information provided. Accurate information of varying degrees of specificity can be provided by offering fact sheets, explanatory leaflets, discussion, counselling, and time to absorb further information (O’Neill 2003).

Although information was seemingly provided in this manner, with multiple information leaflets and the possibility of contacting UK Biobank for more information, something has still gone awry.^8^ Focusing on the idea of informational control, it seems that for participants to have exhibited such control, they would have needed to know what kind of examples of research to further enquire about. That is, participants would have had to construct their own hypothetical research access cases – a process that requires familiarity with a range of industries and the intricacies of the scientific method as applied to health research – in order to glean helpful insights into the proper range of lawful commercial use of their data. Only in doing so could they gain meaningful control over the information they receive and satisfy the consent requirement. This seems out of reach to most laypersons and plausibly places undue onus on the participant to gain information that they deem to be relevant to their consent. It seems, therefore, that the control element stipulated by O’Neill was unattainable in such a scenario, further indicating the possibility of information failures.

In other work, O’Neill notes that consent is a propositional attitude (O’Neill 2001). That is, consent is always directed to some description of a proposal, situation or action. Where a proposition consented to *misdescribes* a proposed action, or is economical with the truth, consent may be misdirected and so will not legitimate. Therefore, consent to a proposition that posits health-research as disease and treatment-focused, therefore, does not transfer to propositions that posit health-research in more encompassing ways, including say, research by insurance companies undertaking risk analysis. Insurance companies’ (lawful) use of UK Biobank data turned out to generate public outcry (Das 2023), which serves to affirm this discrepancy between the propositional content of the consent forms and the full lawful use, and the respective propositional attitudes. In practice, the full scope of use has not been adequately understood by participants and the public, corroborating my analysis. The information leaflet states that “insurance companies and employers will not be given any individual’s information, samples or test result…” (UK Biobank 2010). This claim was seemingly misunderstood as meaning that insurance companies would not have access to UK biobank data at all, but what it really meant was that no identifiable, individual participant data would be shared with an insurance company.

UK Biobank responded to this by claiming that participants had given their consent for their data to be used by approved researchers from all types of academic and commercial organisations for health-related research that is in the public interest (UK Biobank 2023). It claimed that this was made clear to each participant in the information leaflet and the consent form. However, as my earlier analysis suggests, the examples there focus on treatment-based commercial use, which may plausibly have generated the (false) impression that this was the only type of commercial use permitted. Giving examples of different kinds of commercial use, including by insurance companies, could have generated a more holistic impression of lawful use at the informed consent stage. This is particularly troubling given that respondents to a consultation on UK Biobank’s early Ethics and Governance Framework highlighted the need for a clear explanation of what consenting to participate in UK Biobank would entail (UK Biobank 2004). A number of respondents were explicitly concerned about permission being granted for specific types of research, particularly medical market research and insurance industry research (UK Biobank 2004). Indeed, legal work in this area has recognised that participant concerns over control over data may be unrelated to ‘re-identifiability’ and are instead about uses of research that are against one’s ethical beliefs as well as about “possible actors involved in the use of data (insurances, private companies etc)” (Staunton et al. 2019; Caulfield et al. 2014). If participants *truly* understood that their data might be used for purposes that they regard as immoral or repugnant, a broad consent policy may be justifiable (Häyry et al. 2007). The problem here lies, however, in the lack of such transparency and the limited provision of examples that obscure controversial uses.

It is also worth noting that the activities of insurance companies may in fact fall outside of UK Biobank’s stated purpose altogether. Indeed, participants may reasonably question whether the work of such companies is pertinent to disease, treatment, or to the promotion of the health and wellbeing of society. It has been convincingly argued that research undertaken by tobacco companies should not be supported by UK Biobank (Capps and van der Eijk 2014). One might make an argument by analogy in the case of insurance companies. Benjamin Capps and Yvette Van der Eijk suggest that the strategic purpose of the tobacco industry is something that a public resource, such as UK Biobank, should not support. This is because tobacco industry research has been known to work irreconcilably with the public health purposes of institutions like UK Biobank. The tobacco industry has vested research agendas and has previously sought to undermine effective tobacco control policies, obfuscated scientific counterevidence, and exaggerated the findings of its own research (Capps and van der Eijk 2014). In a “Frequently Asked Questions” (FAQ) document that seems to have been removed from the UK Biobank website since Capps et al. accessed it to write their paper, UK Biobank itself states that it is “virtually impossible” to see that an application by the tobacco industry to use the Resource would fulfil the requirements that researchers are bona fide health research scientists and that their work is for the public good. As such, “applications by researchers funded by the tobacco industry (directly or indirectly) would be similarly unlikely to be approved” (Capps and van der Eijk 2014).

Insurance companies, I suggest, also have strategic purposes that may be at odds with the public interest. Life and health insurance companies have an interest in genetic testing as the data derived can be fed into risk assessments and premium calculations. If at-risk individuals are aware of, and withhold, such genetic information, they can seek out insurance at premiums “which do not reflect the extra risk” (Our Future Health 2025). In deciding whether to sell life insurance policies and at what price, insurers routinely consider applicants’ risk factors such as smoking and obesity. They are similarly interested in genetic associations for disease risk (Klitzman et al. 2014), despite such testing being an unreliable indicator for disease (Hingorani et al. 2023; Rappaport 2016). Nevertheless, gaining such genetic information remains appealing for insurance companies. It makes sense, within their strategic purposes, that they would be interested in research into genetic variants and disease. However, the specific strategic purpose of charging a “genetic risk-attentive” premium for insurance in a manner continuous with profit-maximisation is arguably at odds with the public interest. Insurers having widespread access to genetic information may be harmful if it leads to a state of affairs where individuals are compelled to provide genetic test results to them. Those who are found to be at high genetic risk of death and disease may face discrimination on the insurance market. They may be charged unaffordable premiums or be turned away from certain insurers who do not want to attract a disproportionate share of “unhealthy individuals” as this would undermine the stability of the risk pool. Normally, the higher costs of the less healthy would be offset by the relatively lower costs of the healthy. However, the introduction of genetic risk information means that insurers, equipped with this new knowledge, may become more selective about entry into their pools. This could complicate the ability to deliver insurance in a fair and equitable manner and undermine its value as a safety net for the vulnerable. Further, the introduction of genetic testing and assessments may have negative effects on the principle of solidarity and the willingness people display towards pooling risk with each other. Such a situation could arise via the gradual encroachment into genetic research by the insurance industry, a process that has been inadvertently facilitated by UK Biobank in its recent access approvals.

UK Biobank has maintained that insurance companies are bona fide researchers acting in the public interest. It characterises research being undertaken by insurance companies in the following manner: “research that involves more accurate identification, calibration and assessment of the risk of some particular disease would clearly be consistent with being health-related and in the public interest. Likewise, so too would be research that identified certain types of behaviour which increased risk, and so might be avoidable” (UK Biobank 2023). These claims do not address the downstream consequences of insurance companies’ access to genetic information that I have set out above, however. It remains that the routine access to genetic data by insurance companies may lead to a situation in which such companies make the case that genetic risk for disease ought to be factored into their risk-assessments and premium calculations, leading to the dissolution of the social value of insurance hypothesised above. While this may seem conjectural, life insurance companies in the U.S., for example, have already started demanding consumers’ genetic tests and declining entry into their pools on this basis (Brown 2024).

It is now worth returning to UK Biobank’s safeguarding procedures and Widdows’ prescient remarks surrounding their importance in upholding trust and ensuring the terms of consent are not broken. If, as I have suggested, some of the permitted research studies are outside of the biobank’s stated aims, or are not expected by participants, the relevant safeguarding procedures may exhibit fault. These are the EGC (now Ethics Advisory Committee) and the Access Committee. To recall Widdows’ warning, “the trust model improves upon broad consent only to the extent that its ethics and governance mechanisms are robust” (Widdows 2013). The EGC, even while it was still independent had been criticised for being “toothless” and unable to deliver real intervention (McHale 2011). Taking its role in-house, via the Ethics Advisory Committee, may have served to exacerbate these concerns. Individuals involved with UK Biobank have publicly stated the following in a research paper:

All access applications are discussed and approved by the Access Sub-Committee (ASC) of the UK Biobank Board. Access to data is relatively permissive, and review by the ASC seeks only to ensure that the research is viable and meets the requirements. The ASC’s main responsibility is making strategic access decisions, particularly regarding contentious matters and the use of biological samples. Ethics advice is provided to the ASC on an independent consultancy basis by Oxford University’s Ethox group (Conroy et al. 2019).

By its own admission, the approval process is relatively permissive. It is not clear whether the ethics advice is provided ad hoc or routinely. Nevertheless, one can surmise, given all that I have suggested about the consent process and certain uses of research, that there may have been some oversights in this regard. However, even a sufficiently attentive ethics approval process may not have been able to evade the dual use issues that can arise with such research, as I will show in the next section.

Thus far it has been suggested that broad consent given by participants may not have been meaningful. The framing of health-research in the public interest in terms of treatment of disease and health-promotion does not suitably delineate the legal scope for use. There are two implications of my analysis to demarcate: the first is that consent may not have been meaningful if there are information failures resulting from this framing, as certain lawful uses of biobank data may not have been sufficiently understood. The second is that on UK Biobank’s own outlining of research, some of the permitted research studies may turn out to be outside of its stated aims. In this case, relevant safeguarding procedures may exhibit fault, and the terms of consent may have been broken even if it were deemed valid from the outset. I will now turn to show that while certain uses of data may have initially presented as satisfying UK Biobank’s data use-legitimating grounds centred on health research in the public interest, they have gone on to have objectionable further use.

## Data processing and dual use

The so-called “dual-use dilemma” arises in the context of research in the biological and other sciences when the same piece of scientific research has the potential to be used for harm as well as for good (Miller and Selgelid 2007; Parliamentary Office of Science and Technology, July 2009). It is often unclear how to prevent the misuse of such research without foregoing its beneficial applications. Misuse may involve the deployment of scientific knowledge in civilian or military settings to cause unjustifiable harm. The paradigmatic example is advancement in DNA synthesis, which has enabled cheap and rapid synthesis of some viral genomes. This research furthers our understanding of a virus’ properties, equipping us to be better protected from, or treat, human viral disease (Wimmer et al. 2009). However, further advances and improved methods for developing infectious agents may make bioweapons production available to “hostile actors”. The dual-use dilemma is an ethical dilemma for the researcher as well as for regulators who can intervene in the researcher’s work. In this case, UK Biobank and its ethics approval system have the relevant power at the approval of access stage. Researchers themselves should also be cognisant of possible harmful applications of their research. In this section, I suggest that certain uses of UK Biobank data may initially present as permissible but go on to have an objectionable “dual use” that would not be in the public interest. Further, the fast-paced nature of genetic research and the commercial interests that accompany it, seem to facilitate and proliferate the possibility of such dual use dilemmas. Risks associated with dual use are not adequately safeguarded against under the current approach, and it is not clear how they could be without greater restriction of research.

Multiple researchers have accessed UK Biobank datasets to undertake research on the genetic basis of cognitive ability. Dual use issues arise with certain applications of this research. After accessing UK Biobank data in 2015 to investigate the “genetic architecture” of complex traits such as “height, BMI, cognitive ability” (UK Biobank 2015), one researcher went on to co-found a U.S.-based assisted reproductive technology company that applies polygenic risk score analysis on embryos (LeMieux 2019). This is done through in vitro fertilisation and pre-implantation genetic testing (Forzano et al. 2022). To develop this tool, researchers used advanced machine learning methods to analyse almost the entire UK Biobank cohort. Through this process of being trained on the biobank datasets, an algorithm identified gene locations linked to genetic traits and created a predictor (Lello et al. 2018). The researchers claim to have produced “for the first time, accurate genomic predictors for complex traits such as height, bone density, and educational attainment” (Lello et al. 2018). Such a tool, they state, could be used to predict health risks or to create personalised drug therapies, as well to screen embryos and create “designer babies” whereby parents could eventually “choose the traits of their baby” (Shaffer 2018). In further research using the UK Biobank datasets, this researcher’s trajectory was made explicit in a conflict of interest statement accompanying a publication (Yong et al. 2020). Their company provides embryo screening services for those pursuing IVF to “reduce the risk of miscarriage and genetic disease, increasing the success of fertility treatments.” (Genomic Prediction 2025). While Genomic Prediction previously offered to screen for gene variants associated with “intellectual disability”, it now only offers the service for disease risk, maintaining that height and cognitive ability are too controversial to screen for (Ball 2021). A surreptitious investigation into another U.S.-based polygenic screening company, Heliospect Genomics, however, found that it has recently claimed to have helped some parents select future children based on genetic predictions of intelligence, stating that their methods could produce a gain of “more than six IQ points” (Shukman et al., 2024a, b). Heliospect also accessed UK Biobank data as an approved researcher in 2023 (UK Biobank 2023; Shukman et al., 2024a, b). So-called ‘genetic enhancement’ technology is out there and available, therefore, and it was developed in part through the use of UK Biobank data. The issues pertaining to dual are live.

To be sure, the point I seek to make here is not that it is objectionable for researchers to draw upon UK Biobank as a public resource and go on to benefit commercially. Access to UK Biobank by commercial institutions is already guaranteed, and we do not expect academics to refrain from fostering any commercial affiliations themselves, more broadly.^9^ The concern is one of dual use. While the initial research may have yielded important insights that were in the public interest, the application of such research to the development of embryo screening tools, in a way that might constitute neo-eugenic focus on creating people with certain “desirable” traits, is less clearly in the public interest. Indeed, it may be argued that the availability of such technology is poised to generate significant harm. While there exists debate on these issues, with some arguing that selection for non-disease traits such as intelligence could be beneficial (Savulescu 2001), the question as to whether there is harm poised to transpire does not need to be entirely settled in order to point to the requirement to take precautions in this regard.

Why, then, might the deployment of embryo screening tools for intelligence be inimical to the public good? There is an important genealogical aspect to consider, in that research into the heritability of intelligence has historically been intertwined with scientific racism and eugenics (Lombardo 2018; Paul, 1998). The idea that we might be able to select for genes for intelligence in embryos is parasitic upon claims surrounding the heritability of ‘intelligence’ as separable from environmental factors – a far from settled scientific question (Sauce and Matzel 2018). Frances Galton made the first attempt at creating a standardised test to determine an “intelligence quotient”, or IQ. He is also viewed as the father of eugenics, having coined the term and advocated eugenic policies such as encouraging the physically and mentally superior members of the population to choose partners with similar traits (Gillham 2001). Galton believed that intelligence was largely a product of heredity and correspondingly that there were racial differences in intelligence. Oppressive social mores imbued his research which fed into the wider development of race science (Nature, 2022; Nicholas Wright Gillham 2001). As well as IQ and heritability having this encumbered history, there remain scientific concerns with the idea that we might locate genes for IQ. Most geneticists now believe that intelligence is a complex trait that is influenced by both genetic and environmental factors (Sauce and Matzel 2018; de Jong 2000) and that genetics does not explain average differences in IQ test performance between groups (Jackson and Weidman, 2004). We ought to be cognisant of the way in which this encumbered history informs the continued advancement of IQ and heritability discourse. Further, we ought to be vigilant of the way in which any such findings into the heritability of intelligence among individuals might fuel research into group-based ‘genetic’ disparities in IQ.

Indeed, this very link is evinced by genomic prediction enthusiasts themselves. Research into a genetic basis of intelligence often goes hand in hand with beliefs surrounding group-based genetic differences in intelligence and in a biological conception of race. Researchers involved with the very embryo screening companies I mentioned that drew upon UK Biobank data, have aligned themselves with the view that races are biologically real (Hsu 2007; Jackson 2020; Shukman et al., 2024a, b) as well as the view that there are plausibly racial differences in intelligence (Winegard et al., 2020). In debates on the metaphysics of race – that is, questions surrounding whether race is best understood as a natural kind or social construct – the historical conception of race treats race as an objective biological kind, grouping human populations according to genetically determined physical and behavioural differences. It has faced significant scientific and philosophical challenge, and there is widespread scholarly consensus that the idea of discrete or essentialist racial groupings is socially constructed, not biologically real (Romualdi et al. 2002).

While advocates of a biological conception of race may claim that nothing normative follows from a descriptive claim that race is biologically real, and that such a ‘reality’ would not justify oppressive treatment, it may be countered that claims about the biological reality of race are not merely descriptive. That is, it the historical, biological conception of race is inextricably tied with the scientific racism that accompanied it and seems inconceivable without the racialised social hierarchies that it was constructed to uphold (Barn 2022). The effects of these social hierarchies linger, and continuing to view race in this way may serve to perpetuate these effects. Indeed, racial essentialism has been found to be associated with increased prejudice toward those racialised as Black among both White and Black adult participants (Mandalaywala et al. 2018) through the belief mechanism that social categories reflect objective structure in nature, and thus that observed social hierarchies and inequalities are also natural.

Jonathan Kaplan et al. argue that “hereditarian” beliefs in “racial” differences in average IQ test performance are predicated upon a systematic denial of the roles played by racism (and race more generally) in society today (Kaplan et al. 2014). This kind of systematic denial not only gives “aid and comfort” to racists and racist positions but *is itself* a kind of racism and is at least evidence of racist intent. This is because such a view minimises and ignores the historical and continued existence of racism and its effects. Indeed, such a link between racist intent and beliefs in genetic bases for group-based behavioural differences was also on display in the recent reporting that individuals from the Human Diversity Foundation (HDF), a group engaged in far-right political activity and the promotion scientific racism, had gained access to UK Biobank data (Shukman et al., 2024a, b; Burgis et al. 2024; Pegg et al. 2024). HDF is an extremist organisation that endorses the idea of creating a White ethnostate by a process it terms “remigration”, involving forcibly expelling non-ethnically European minorities (Shukman et al., 2024a, b). Members of HDF were covertly filmed discussing the fact that they had gained access to UK Biobank genetic datasets that they were “not meant to have” (Burgis et al. 2024). In theory, machine learning tools could be used to analyse these data sets and find associations between ‘race’ (understood as the ethnic backgrounds disclosed by participants) and cognitive ability (generated using the cognitive tests conducted by the biobank or educational level as a proxy), with the aim of using this research towards political ends^10^ (Richardson 2011). UK Biobank responded that claims of a data breach were unfounded, and that “none of the individuals named by *The Guardian* have ever been approved for access”(UK Biobank 2024). This does not extinguish the possibility that an approved researcher directly passed on the data, an eventuality that was explicitly outlined by one HDF researcher: “the only way we get these datasets is when some academic gets them and gives them to us under the table…not necessarily academics, sometimes private sector … taking a big risk for themselves” (Burgis et al. 2024). In order to enhance data governance and safeguard against such unauthorised access, UK Biobank could consider using secure data access environments like the Kapseli (“Capsule”) implemented by Findata in Finland (“Kapseli^®^,” 2025) This is a secure and remote data processing environment that prevents data export.^11^

Clearly the use of data towards the ends of far-right extremism and “race science” fall beyond the original scope for use and thus breach the lawful grounds for data processing.^12^ Indeed, this was recognised by UK Biobank in its response to the potential breach: “when our participants joined UK Biobank 15 years ago, they agreed that their health data could be used for health research in the public interest. This means the findings should benefit the health and wellbeing of society. The findings should not cause harm, such as perpetuate stereotypes about certain groups, and we do not allow researchers to use UK Biobank data for so-called ‘race science’”(UK Biobank 2024). On the one hand, therefore, UK Biobank recognises that research into race science would not be in the public interest. On the other, it permits its datasets to be used for genomic predictive services around cognitive ability – research that has reinforcing effects on race science, a link that is displayed by the interests of researchers in this area themselves.

Even if one were sceptical of these race-based societal harms, it remains that such tools have been developed using the data of people who did not anticipate this dual use, which further troubles UK Biobank’s reliance on broad consent and indeed extends beyond this, given that objectionable downstream uses can be unforeseeable at the time of consent. Participants may have been unlikely to consent to this specific use of their data, for various moral and sociopolitical reasons. Further, as my earlier analysis of consent suggests, participants enter this study expecting their data to be processed towards research that focuses on health understood as treatment for disease. They are not expecting uses centred on controversial non-disease expansions of the genetic screening industry, even if such uses of their data can be said to be lawful. The fact that such data processing occurs, which seems so detached and dissimilar from the examples provided at the consent stage, further emphasises the fact that the consent process fails to give due legitimacy to various *de facto* uses of UK Biobank. Such cases reinforce my claim that participants fail to be fully informed about the range of legally permissible uses of their data. This concern is particularly resounding and finds force when viewed in light of how the data is being used in actual fact.

It is also worth noting that the fast-paced nature of the tech industry coupled with the lack of governance and democratic oversight, particularly in deregulated environments like the U. S., mean that dual use concerns are proliferated. It may be helpful here to draw an analogy with the idea of open-source code. There exist various software and hardware projects under open licenses that allow anyone to build, customise, and experiment with their own drone devices, for example. This phenomenon opens up the public to various risks, including privacy harms if cameras are installed on drone devices. UK Biobank, although not quite “open source”, has arguably been accessible in a way that has generated downstream objectionable uses and risk of harm, in a way that was not anticipated when it was set up as an accessible resource. One crucial point of disanalogy, however, which serves to illuminate the problem even further, is that open-source code for drones did not rely upon the voluntary participation of half a million individuals who thought they were donating personal data for the good of public health. Greater restriction into the processing of UK Biobank data may be required to mitigate against such dual use concerns. However, objectionable further uses of research are hard to anticipate at the point of approval. It is not clear that greater ethical oversight over the research approval process would help. Again, the possibility of unforeseen and objectionable uses of permitted research ought to be outlined to participants at the ‘broad consent’ to participation stage, and now relayed to all via personal communication, at the very least.

## Concluding remarks

I have suggested that UK Biobank’s framing of health-research in the public interest in terms of treatment of disease and health-promotion does not suitably delineate the legal scope for use. As a result of this, participants may not expect their data to be processed by insurance companies and direct-to-consumer genetic testing companies. The inclusion of additional examples in UK Biobank information leaflets that stipulate these controversial uses could help mitigate this by more accurately reflecting the range of future data uses. However, the use of biobank datasets towards the ends of insurance and genetic ancestry companies may fall outside the bounds of health-research in the public interest altogether. I have aimed to provide a preliminary argument to this end with the case of insurance companies’ access. Such wrongful uses call into question the effectiveness of various safeguards, including the role of the UK Biobank’s Access Sub-Committee. While it is beyond the scope of this paper to deliver any all-things-considered recommendations, it may be suggested that UK Biobank reconsiders its data access policy in order to ensure stronger alignment with the public interest standard it seeks to uphold. The restriction of research in accordance with democratic values and the communal good would be consistent with public interest and scientific research purposes, appropriately conceived. Further, UK Biobank must work to regain any possible loss of trust that results from these controversial applications of its data. This is imperative to its survival: often, willingness to participate in research is based on trust in specific institutions (Caulfield et al. 2014). Several studies suggest that many participants display negative attitudes towards the involvement of commercial entities in biobanks and biobanking research (Caulfield et al. 2012; Nilstun and Hermerén 2006; Lemke et al. 2010), and this is quite apart from the tangible and specific controversies I have described above.

I have also made the case that approved biobank research can go on to have further, objectionable uses that fall outside the public interest and may be contrary to the health and wellbeing of society. The fast-paced nature of AI-assisted genetic research and the commercial interests that accompany it seem to facilitate and proliferate the possibility of such dual use issues. Risks associated with dual use are not adequately safeguarded against under the permissive public interest-based GDPR grounds for data processing and it is not clear how they could be without the greater restriction of research suggested above. At least two genomic prediction companies have used UK Biobank data to build their polygenic risk score mechanisms which they may be offering to consumers of their IVF services to screen embryos for non-disease traits such as “intelligence.” These tools rely on contested assumptions surrounding the heritability of intelligence, an area of research that is deeply intertwined with scientific racism and eugenics. Participants to UK Biobank may feel concerned, and possibly even misled, upon the realisation that their donations for health research have contributed to this field, which plainly falls outside of the public interest and seems remarkably detached from the informed consent documents they signed.

## Footnotes

1 In late 2023, Schmidt and Griffin were announced as the first members of a consortium that would “shape the future” of UK Biobank. They jointly donated £16 million to support the growth of UK Biobank’s data in order to unlock “the next great leaps in our understanding of health and disease.”

2 While this may raise concerns regarding the commercialisation of genetic material and profit-sharing, this will not be my focus here.

3 These safeguards do not mitigate against the possibility of unforeseen and dual use of research that will be discussed later, however.

4 I am grateful to an anonymous reviewer for this observation.

5 (“Consent Form: UK Biobank,” 2006) Emphasis added.

6 (“Consent Form: UK Biobank,” 2006) Emphasis added.

7 (“Consent Form: UK Biobank,” 2006) Emphasis added.

8 O’Neill also stipulates that the information provided to patients must be accurate. It may be argued that the information provided to UK Biobank participants failed to meet accuracy requirements due to the limited examples of use given and their treatment-focused nature. I will set aside accuracy concerns here and assume that the information provided was accurate but that there remained a of lack of control that contributed to information failures.

9 Although arguments can certainly be provided as to why such affiliations might inhibit academic freedom.

10 Appeals to “intelligence” have historically sanctioned the sterilisation, murder, and incarceration of human beings that society deems disposable. Intelligence tests were also deployed as part of early 20 th Century American immigration policy to turn away “feeble-minded immigrants.”

11 I am grateful to an anonymous reviewer for this helpful example of alternative data governance solutions.

12 As a result, participants may want to withdraw their data from the dataset and prevent any further processing.
